# Ozone therapy as a novel complementary therapeutic approach in refractory idiopathic granulomatous mastitis

**DOI:** 10.3389/fmed.2023.1174372

**Published:** 2023-07-06

**Authors:** Neslihan Cabioglu, Didem Can Trabulus, Selman Emiroglu, Enver Ozkurt, Nesli Yalcin, Nagehan Dinc, Mustafa Tukenmez, Mahmut Muslumanoglu, Abdullah Igci, Vahit Ozmen, Ahmet Sait Dinccag, Yusuf Izzettin Guven

**Affiliations:** ^1^Istanbul Faculty of Medicine, Department of Surgery, Istanbul University, Istanbul, Türkiye; ^2^Department of Surgery, Bahcesehir University, Istanbul, Türkiye; ^3^Department of Surgery, Istanbul Florence Nightingale Hospital, Istanbul, Türkiye; ^4^Department of Surgery, American Hospital, Istanbul, Türkiye; ^5^Continuing Medical Education, Medipol University, Istanbul, Türkiye

**Keywords:** idiopathic granulomatous mastitis, ozone therapy, steroid, refractory granulomatous mastitis, steroid resistance

## Abstract

**Background:**

Autoimmunity may play a major role in the pathogenesis of idiopathic granulomatous mastitis (IGM). The therapeutic potential of ozone therapy has recently been shown in rheumatological diseases, and this study aimed to assess the clinical efficacy of ozone therapy (OT) in refractory IGM.

**Methods:**

Patients with biopsy-verified IGM and incomplete response after steroid therapy (*n* = 47) between 2018 and 2021 were enrolled. Of these, 23 cases in cohort A had standard treatment with further steroid therapy (ST), and 24 were treated with systemic OT via autohemotherapy (AHT) in addition to steroid therapy (cohort B).

**Results:**

The median age was 33 years (range, 24–45). Patients in cohort B had a higher complete response rate after completion of a four-month ozone therapy than those in the ST-group (OT-group, 37.5% vs. ST-group, 0%; *p* = 0.002). At a median follow-up of 12 months (range, 12–35), the patients treated with OT had a lower one-year recurrence in the affected breast than cases in cohort A treated with ST (OT-group, 21% vs. ST-group, 70%; *p* = 0.001). No significant side effects were observed in patients in cohort B related to AHT. Furthermore, OT significantly decreased the total steroid treatment duration (median week of steroid use; 26  weeks in cohort A vs. 12 weeks in cohort B; *p* = 0.001).

**Conclusion:**

Systemic OT increases the complete response rate and decreases the duration of steroid treatment in patients with refractory IGM. Therefore, ozone therapy is an effective, well-tolerated, and safe novel complementary therapeutic modality.

## Introduction

Idiopathic granulomatous mastitis (IGM) was first described by Kessler and Wolloch as a benign inflammatory breast disease that can mimic breast cancer both clinically and radiologically (1, 2). Histologically, IGM is a chronic granulomatous inflammation of the breast, but there is a lack of information about the real etiology and pathology behind this mastitis (2). One theory is an autoimmune reaction to the secretion extravasated from lobules (2). Unexpected findings include the large number of cases without accompanying autoimmune disease, rare detection of vasculitis, plasma cell infiltration upon histologic examination, and very few cases presenting antinuclear antibodies and/or rheumatoid factors (3, 4). Recent studies demonstrating immunologic dysregulation as the underlying etiology have inspired other studies focusing on detailed immunologic analysis of IGM (5, 6).

Granuloma-forming pathologies should be excluded to define a mastitis as IGM, e.g., infectious diseases such as tuberculosis, systemic diseases like sarcoidosis, and rheumatologic diseases such as systemic lupus erythematosus. There is still no consensus on the effective treatment of IGM. Most common therapeutic approaches are systemic and/or topical or intralesional corticosteroid administration and surgery (7–16). However, these treatment modalities are not highly effective for recurrent or treatment-resistant cases.

There are many experimental and clinical studies about the therapeutic effect of systemic and/or local ozone therapy (OT) on wound healing, rheumatological diseases, and other autoimmune diseases (17–25). However, there are also few studies using OT on mastitis. All of these prior studies used animal models such as cows (26–29). Surprisingly, there is no research on the efficacy of OT on human mastitis. We have recently shown that low-dose OT can have an immunomodulatory effect on patients with refractory IGM (30). In this clinical study, we investigated the clinical efficacy of OT in patients with refractory IGM treated with steroids compared to those patients treated with steroids alone.

## Methods

This study was approved by the Ethical Committee of the Medipol University and the Turkish Health Ministry. Informed consent was obtained from all patients at the University of Istanbul, Istanbul Faculty of Medicine, Breast Surgery Service at the Department of General Surgery. The prospectively-maintained data was retrospectively analyzed and included two cohorts: cohort A (*n* = 23) and cohort B (*n* = 24). These cohorts were treated with two different treatment protocols between March 2018 and March 2021, respectively. Patients complaining about breast mass, inflammation, and pain were evaluated by radiological imaging to exclude any malignancy and for definitive diagnosis. All patients had an ultrasound (USG). Of those, subjects ≥35 years also had a MMG (38.3%, *n* = 18) on first admission.

### Inclusion and exclusion criteria

All patients >18 years of age and with a pathologic diagnosis of granulomatous mastitis were included. The definitive diagnosis was made by core biopsy in all patients. Pathologic examination revealed an inflammatory reaction disrupting the breast lobules with infiltrating epithelioid histiocytes, multinucleated Langhans-type giant cells, lymphocytes, polymorphonuclear leucocytes, and plasma cells as non-caseating granulomas characterized as chronic lobulitis.

All patients had a negative Quantiferon®-TB Gold test, and negative microbiological cultures or reverse transcriptase-polymerase chain reaction (RT-PCR) for tuberculosis that was performed on pus samples obtained from patients with cutaneous fistulas. Severe IGM was defined as the presence of multifocal or multicentric painful granulomas with either abscess or cutaneous fistula formation with/without discharge and erythema, bilateral disease, and/or systemic signs such as erythema nodosum.

All patients who received steroid therapy started on doses from 8 mg to 32 mg methyl prednisolone per day for a median of 24 weeks (range, 8–36). Patients with residual granulomas despite at least 4 weeks of steroid use were included. Resistance to steroid therapy was defined as having residual painful inflammatory granulomatous lesions with abscess or cutaneous fistulas despite oral steroid use for at least 1 month.

The exclusion criteria included contraindications for ozone therapy: pregnancy, hyperthyroidism if not controlled, and coexisting hematologic malignancies (leukemia, etc). Other exclusion criteria include a glucose-6-phosphate dehydrogenase deficiency that could be associated with hematological disorders (favism, acute hemolytic anemia) as described previously (31, 32).

### Application of ozone therapy

Treatment included autohaemotherapy (AHT) in Cohort B (*n* = 24) treated with OT. A Dr. Hänsler Ozonosan Photonic® device (Dr. J. Hänsler GmbH OZONOSAN, Germany) was used to obtain ozone gas. The ozone bottle and set were disposable and fully ozone compatible (Medipac GmbH, Germany). Patients were exposed to an oxygen-ozone mixture at a normalized ozone concentration between 15 μg/NmL (μg/NmL = Microgram/Normalized milliliter of ozone concentration) to 30 μg/NmL. The number of treatment sessions and the ozone dosage administered depend on the general condition of the patient, and course of the disease. The OT was started with the highest dose as 30 μg/NmL in the first 2 weeks, which is considered as an immunesupressive dose, and the dose was deescalated to 15 μg/NmL in regards to the clinical recovery in the following weeks.

During major autohaemotherapy (MAH), 100 cc of blood was preferentially withdrawn from an antecubital vein and mixed gently with 100 cc O3 gas at a 1:1 ratio of oxygen-ozone to blood volume for at least 5 min as previously described (33–37). Briefly, 100 mL of blood was withdrawn from each patient into special ozone bottles under vacuum containing 12 cc 3.13% sodium citrate. The ozonized blood was then reinfused intravenously in the same vein over 10–15 min in concordance with the recommendations of the World Federation of Ozone and the recent Madrid Declaration (35, 36). Patients also received minor autohaemotherapy (MiAH) in addition to MAH. MiAH involves mixing venous blood from the patient (5 mL-10 mL) drawn into a syringe without anticoagulant with the ozone (syringe already contains the ozone-oxygen mixture at 10 μg/NmL to 40 μg/NmL). The blood and ozone mixture is then shaken and reinjected intramuscularly (36).

Our standard approach was to perform OT twice a week for the first month and weekly for the second month followed by the maintenance therapy sessions following full recovery of the breast. The maintenance therapy was generally deescalated ranging from 1 to 4 sessions per month for the first 2 to 6 months, and repeated every 6 months for the first 2 years with the aim to maintain the potential immunomodulation that could prevent the IGM recurrences (30). The de-escalation of the OT sessions was modified according to the clinical course of each patient, and according to our clinical observational experience in OT (unpublished observation).

### Measurement of clinical response

The patients’ demographic features, complaints, imaging methods in diagnosis, pathology results, treatment agents, and duration of treatments were recorded. Patients were clinically evaluated before and after the therapy. The clinical response was mainly evaluated by physical exam and clinical recovery. Radiology including ultrasound or magnetic resonance imaging before and after the therapy were also performed in some patients to assess the clinical trajectory. Briefly, a partial response and minimal response were defined as disappearance of clinical symptoms and inflammatory lesions ≥50 and < 50% following treatment, respectively. A complete clinical response was defined as a complete disappearance of complaints including pain and discharge from fistulas and inflammatory signs in breast including closure of fistula orifices and/or skin erosions. Recurrence was defined as relapse of symptoms after completion of treatment. Outcomes were evaluated by any recurrence in the affected or contralateral breast at 1 year as determined according to the median follow-up time of each cohort. A partial response was defined as disappearance of inflammatory lesions and clinical symptoms ≥50% by physical exam.

### Statistical analysis

Statistical software SPSS 25 was used for analyzes (Statistical Package for Social Sciences; SPSS, IBM Corp., Armonk, NY, United States). Categorical variables were evaluated by Fisher’s Exact Tests in two-tailed univariate analyzes to estimate the differences between the groups; a Mann–Whitney U test was used to determine the differences between continuous variables. A value of p equal or less than 0.05 was considered to be statistically significant.

## Results

Between March 2018 to March 2021, 47 patients were included into the study. Patient demographic characteristics and physical examination findings are shown in Table 1. The median age was 33 (range, 24–45), and the median number of pregnancies was two (range, one to five). The median body mass index was 26.6 (range, 19.2–35) indicating that most patients were overweight or obese.

**Table 1:** Demographic and clinical characteristics of patients with idiopathic granulomatous mastitis with previous steroid use.

Patient characteristics	Total (*N* = 47)	Cohort A (*n* = 23)	Cohort B (*n* = 24)	Value of *p*
Median age (range, min-max)	33 (24–45)	32 (24–45)	34 (28–45)	0.281
First menstrual age	13 (11–17)	13 (11–16)	13 (11–17)	0.778
Nulliparous	0% (0/47)	0% (0/23)	0% (0/24)	0.999
Median first parity age	25 (18–37)	26 (18–30)	24 (18–37)	0.172
Median number of births	2 (1–5)	2 (1–5)	2 (1–4)	0.389
Smoking	10.6% (5/47)	8.7% (2/23)	12.5% (3/24)	0.999
Median body mass index	26.6 (19.2–35)	26.6 (19.2–35)	26.7 (20.4–32.3)	0.870
Family history of breast cancer	14.9% (7/47)	13% (3/23)	16.7% (4/24)	0.999
Education high*	27.7% (13/47)	21.7% (5/23)	33.3% (8/24)	0.517
Bilaterality	12.8% (6/47)	8.7% (2/23)	16.6% (4/24)	0.666
Mass	100% (47/47)	100% (23/23)	100% (24/24)	0.999
Erythema	100% (47/47)	100% (23/23)	100% (24/24)	0.999
Pain	100% (47/47)	100% (23/23)	100% (24/24)	0.999
Erythema nodosum	8.5% (4/47)	8.7% (2/23)	8.3% (2/24)	0.999
Multifocality/ Multicentricity	78.7% (37/47)	82.6% (19/23)	75% (18/24)	0.724
Abscess formation	97.9% (46/47)	100% (23/23)	95.8% (23/24)	0.999
Discharge	97.9% (46/47)	100% (23/23)	95.8% (23/24)	0.999
Cutaneous fistula formation	97.9% (46/47)	100% (23/23)	95.8% (23/24)	0.999
The median size of the granuloma	45 mm (range, 20–100 mm).	40 mm (range, 20–100 mm).	46.5 mm (range, 20–70 mm).	0.192

*Mann–Whitney U-test was used. N.A. = not applicable.

Six patients (12.8%) had bilateral disease, and 8.5% had erythema nodosum. All patients presented with inflammatory lesions with pain, erythema, and mass (*n* = 47, 100%). Almost all patients presented with abscess formation, cutaneous fistula formation, and discharge (*n* = 46, 89.4%). The median size of the inflammatory lesion was 45 mm (range, 20–100 mm). Most patients (*n* = 37, 78.7%) were diagnosed with multifocal or multicentric lesions. No significant differences were seen in the demographic and clinical characteristics between patients in cohort A and B (Table 1).

### Treatment and outcomes

**Figure 1:**
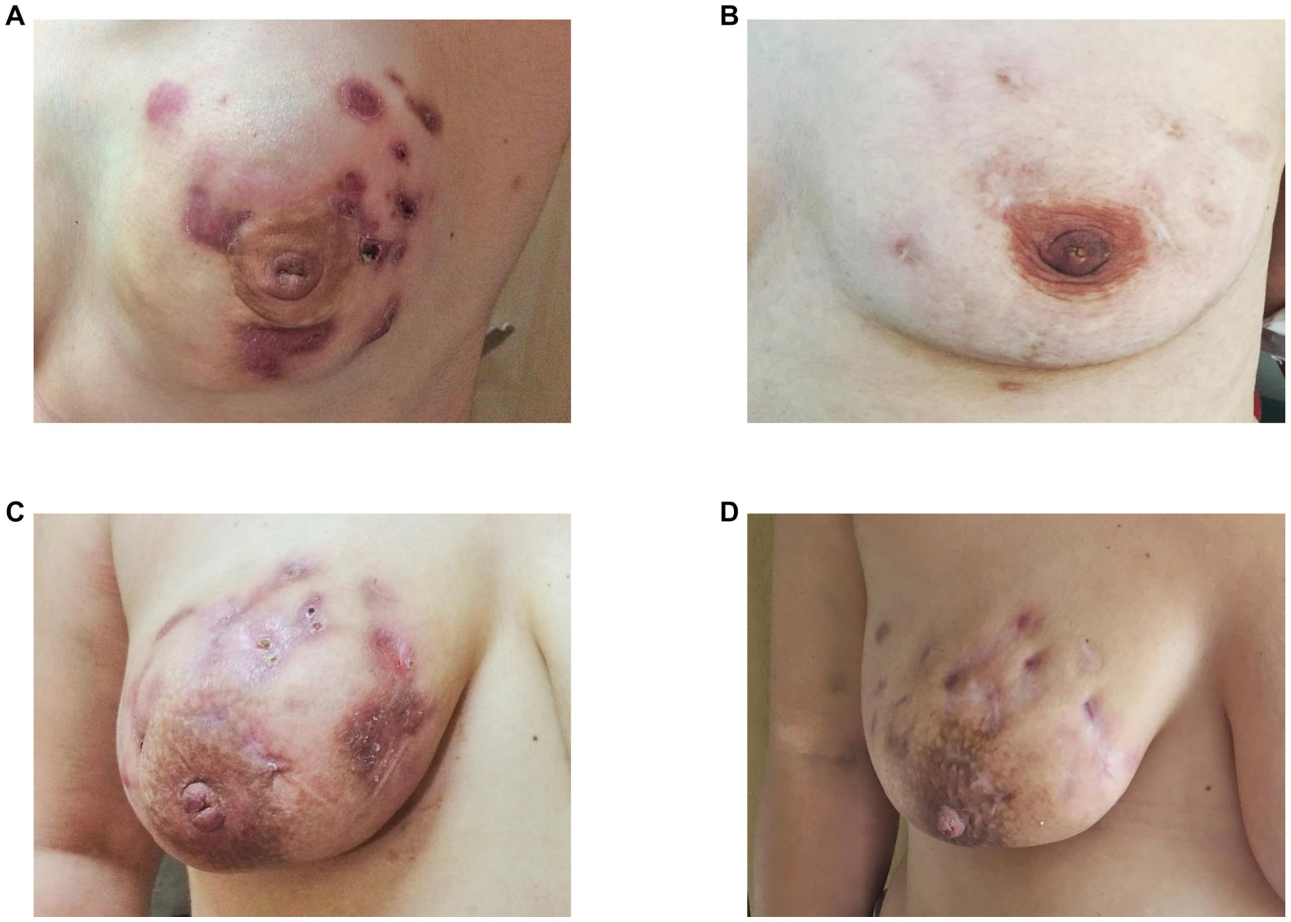
Complete **(B)** and partial **(D)** clinical remission of idiopathic granulomatous mastitis following completion of ozone therapy as seen in two patients as disappearance **(B)** or partial regression **(D)** of abscess and cutaneous fistula formation, erythema, and oedema before **(A,B)** and after ozone therapy **(C,D)**, respectively.

**Figure 2:**
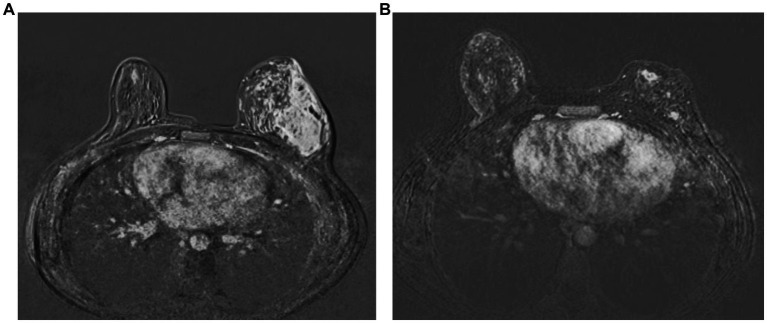
Complete radiological response following completion of ozone therapy as seen in a patient as disappearance of abscess formations and rim-like enhancements and diffuse oedema in contrast-enhanced subtraction images in MRI before **(A)** and after ozone therapy **(B)**, respectively.

Of the 47 patients, patients in the ozone therapy group had already received oral corticosteroids before beginning ozone therapy. These patients were in this study because they did not show a sufficient clinical response to steroids. Cortisone therapy was gradually discontinued after initiation of ozone therapy. The median ozone therapy session was 16 (range, 10–25). Significant clinical remission was seen in all patients following ozone therapy, i.e., softening of inflamed breast tissue as well as a reduction and total disappearance of discharge from cutaneous fistulas in all patients (Figure 1). More than half of the patients (61.7%, *n* = 29) had an MRI showing clinical healing corresponding to decreased breast inflammation following completion of ozone therapy (Figure 2).

Most patients (91.7%) had a complete or partial response following ozone therapy. Only few patients (8.3%) had a minimal response following ozone therapy. A complete response rate after ozone therapy was seen in nine of the 24 patients (37.5%) in cohort B. None of the patients with standard treatment with further steroid use had a complete response in cohort A (Table 2). The median follow-up was 12 months (range, 12–35 months). Patients treated with ozone therapy had a lower one-year and two-year recurrence rate estimated as 21 and 29% in cohort B; those with standard treatment in cohort A had recurrence rates between 70 and 74% (*p* = 0.001 and *p* = 0.003, respectively). Furthermore, ozone therapy significantly decreased the total median duration of steroid use as shown in Table 2 (Cohort A: 26 weeks vs. Cohort B: 12 weeks, *p* = 0.001). No significant side effects were observed in patients treated with ozone therapy.

**Table 2:** Treatment characteristics and treatment response in Cohort A and Cohort B treated with standard therapy (oral steroids) and ozone therapy.

Patient characteristics	Cohort A (Steroid therapy only) (*n* = 23)	Cohort B (Steroid therapy and ozone therapy) (*n* = 24)	Value of *p*
Median follow-up time (months)	12 (range, 12–35)	13 (range, 12–23)	0.447*
Number of ozone applications	NA	16 (range, 10–25)	
Median week of steroid use	26 (range, 16–36)	12 (range, 8–32)	0.001
Median maximum methyl prednisolone dose per day	16 mg (range, 8–32)	16 mg (range, 8–32)	0.650
Response to four-month therapy after diagnosis			**0.005**
Complete response rate	0% (0/23)	37.5% (9/24)	
Partial response (>50% disappearance of lesions)	65.2% (15/23)	54.2% (13/24)	
Minimal response (<50% disappearance of lesions)	30.4% (7/23)	8.3% (2/24)	
No response/progression	4.3% (1/23)	0% (0/24)	
Complete response rate	0% (0/23)	37.5% (9/24)	**0.002**
One-year breast recurrence rate	70% (16/23)	21% (5/24)	**0.001**
Two-year breast recurrence rate	73.9% (17/23)	29.2% (7/24)	**0.003**

*Mann–Whitney U-test was used. N.A. = not applicable.

## Discussion

Idiopathic granulomatous mastitis (IGM) is a rare and chronic inflammatory breast disease with an unclear etiology. There is no standard treatment approach due to its elusive etiology. Relapses are common after the end of treatment. The most common modalities are systemic and/or topical or intralesional corticosteroid administration and surgery (7–16) There are limited data about therapy in steroid resistant-patients with IGM.

Kundaktepe et al. (38) recently reported excellent outcomes with MTX monotherapy in 64 patients; 56 of these were resistant cases. Here, we present ozone therapy as a complementary treatment tool. We achieved excellent outcomes with a high complete response rate and low one-year-recurrence rate following ozone therapy in patients with IGM who could not achieve complete or satisfactory partial response after steroid treatment.

The therapeutic effects of systemic and/or local ozone administration have been investigated in different fields of medicine (17–25, 31, 32, 37). Zhang and colleagues investigated the effect of ozone therapy on wound healing (19). They found that ozone alters the levels of growth factors including transforming growth factor beta, platelet-derived growth factor, and vascular endothelial growth factor; these are effective in wound healing. A recent systematic review of 12 clinical studies (18) demonstrated that ozone therapy markedly accelerated wound improvement and reduced the amputation rate versus standard control therapy for diabetic foot ulcers; this was true regardless of monotherapy or combined control treatment [RR = 0.36, 95% CI = (0.24, 0.54), *p* < 0.00001].

Previous studies have shown that ozone therapy reduces inflammation as well as IL-1β and TNF-α mRNA levels; ozone decreases oxidative stress in PG/PS-induced arthritis in rats (23). A randomized trial demonstrated that ozone therapy in addition to MTX treatment improved clinical response versus MTX, folic acid, and ibuprofen therapy in patients with rheumatoid arthritis (RA) (24). Ozone therapy combined with MTX increased the antioxidant system and decreased oxidative damage compared to MTX alone in these patients. These findings suggest that medical ozone treatment is an effective and complementary therapy in RA treatment.

There are few reports about OT in mastitis, and all of them are experimental studies on animal models (26–29). Ogata et al. reported that 60% of the 15 cows with acute clinical mastitis were successfully treated with intramammary ozone application alone without any antibiotic treatment (26). Ohtsuka et al. demonstrated that the CD4(+)/CD8(+) ratio was increased in cows with inflammatory diseases including mastitis (*n* = 4) following systemic OT as an autohaemotherapy (27). The autohaemotherapy included insufflation of a blood volume of 25 to 100 mL with ozone (10 μg/mL) to maintain the overall ozone concentration (20 μg/kg body weight). In contrast, the number of CD14+ and MHC class-II (+) cells were estimated to decrease in the OT-group versus the control group. These findings suggest an immunomodulatory effect of systemic OT via autohaemotherapy in infected cows.

To the best of our knowledge, this is the first study describing the therapeutic efficacy of OT in IGM patients by decreasing the need of steroid use. Systemic ozone application via AHT was tested here as a preliminary study. This work investigated the efficacy of OT in patients with severe IGM resistant to conventional therapies. Of note, our breast surgery service is a tertiary referral center. A significant proportion of our patients are secondary admissions who have previously been treated with steroids; thus, we defined these cases as refractory to conventional therapies. Furthermore, most patients included in this study had severe IGM who presented with painful and multifocal/multicentric inflammatory granulomas with either abscess or the formation of cutaneous fistulas.

We identified significant clinical remission as softening of breast tissue, shrinkage of the affected area, and complete disappearance of discharge from cutaneous fistulas in almost all patients of the cohort treated with OT. Complete remission was obtained in 37.5% of patients treated with OT after 4 months of treatment. Most of the remaining patients had a partial response (54.2%); only a small percentage of patients (8.3%) had a minimal response to low-dose OT. As expected, OT also diminished the total steroid use duration in patients, which might potentially decrease the side effects of steroids. Furthermore, patients treated with OT via AHT had a lower one-year recurrence rate (21%) than patients treated with further high-dose oral steroids (70%).

The two-year breast recurrence of IGM was 29% in the OT-group. There was a trend toward an increased recurrence rate with long-term follow-up. This is probably due to a lack of compliance with maintenance OT during the COVID-19 pandemic. Therefore, the maintenance therapy repeated every 6 months following recovery after an intensive OT may to be critical to achieving long-term clinical responses in these patients. OT should be best considered a continuous therapy and should be applied over certain intervals to prevent the recurrence of IGM as a chronic inflammatory breast disease.

In the present study, patients treated with OT via MAH and MiAH were presented. Our OT protocol always included applying MAH with MiAH, since MiAH may potentially increase the therapeutic efficacy of MAH, as an auto vaccine. OT via different other routes including rectal and/or topical routes is a subject of another investigational study by our research group that will be reported in future.

Similarly, OT can be an effective complementary or alternative therapy in patients with severe IGM resistance to conventional therapies including steroids or MTX. Future studies should also be directed to the underlying mechanisms and therapeutic effects of OT in patients with IGM. Previous studies have shown that low-dose ozone acts as a bioregulator in chronic inflammatory diseases (31, 32). Systemic OT improves the antioxidant capacity and leads to a significant reduction in oxidative stress. Cytokines are modulated via the downregulation of inflammatory cytokines as seen in chronic inflammatory diseases including RA as a model for chronic inflammation. We have recently reported that OT had an immunomodulatory effect on patients with refractory IGM (30). Our results demonstrated that OT stimulated a T-helper-1 response associated with IFN-γ production and downregulation of TGF-β expression in CD4^+^ CD25^+^ CD127-Treg cells. These alterations in the immune system following ozone therapy might be associated with wound healing by restoring immune dysfunction in patients with refractory IGM. Based on previous studies in RA and our findings in IGM, these results confirm that integrating OT as a complementary and/or alternative therapy with conventional therapies might be a promising approach in the treatment of severe resistant IGM.

The current international guidelines strongly suggest using systemic OT (≤40 μg/NmL) via AHT which has been standard in clinical trials with OT (31, 32, 37). Our patient population consists of women of reproductive age without any comorbidities; previous publications have shown that OT (≤40 μg/NmL) appears to be safe in this group (30, 31). No significant side effects were observed in our population treated with the standard doses of OT (≤40 μg/NmL).

Ozone therapy is not covered by Turkish health insurance, and thus it can be an expensive treatment unless included in clinical trials. Therefore, the cost-effectiveness of this treatment modality and the associated impact on quality of life should also be investigated in patients suffering from chronic breast mastitis.

## Conclusion

Our findings suggest that systemic OT for the management of severe refractory IGM is an effective safe novel complementary treatment. Such treatment should not be substituted for standard IGM treatments. Therefore, OT should be best administered in selected patients with refractory disease or severe IGM who do not desire long-term high dose steroid treatment. Ozone is a complementary modality that potentially decreases both the total steroid use duration and the complications associated with steroids. Future randomized controlled trials are required to evaluate the efficacy of OT as a first-line treatment approach in selected patients with severe IGM. The clinical efficacy of OT via different routes, i.e., rectal and/or local intramammary administration should also be studied in further studies.

## Data availability statement

The original contributions presented in the study are included in the article/Supplementary material, further inquiries can be directed to the corresponding author.

## Ethics statement

The studies involving human participants were reviewed and approved by The Institute’s Ethical Committee on Complementary Medicine from the Medipol University on human research. This was further approved by the Turkish Health Ministry (37106781–000-86,033). The patients/participants provided their written informed consent to participate in this study. Written informed consent was obtained from the individual(s) for the publication of any potentially identifiable images or data included in this article.

## Author contributions

NC, YG, and AD designed the study. NC, DT, EO, and SE performed the initial search, literature organization, analyzes, and manuscript writing. NC, DT, NY, SE, MT, MM, AI, YG, and AD provided data acquisition. DT, NY, SE, EO, ND, MT, MM, AI, VO, YG, and AD made the critical comments and typesetting corrections on the final version. All authors have read and revised the manuscript critically.

## Funding

This project was supported by the Istanbul Breast Society.

## Conflict of interest

The authors declare that the research was conducted in the absence of any commercial or financial relationships that could be construed as a potential conflict of interest.

## Publisher’s note

All claims expressed in this article are solely those of the authors and do not necessarily represent those of their affiliated organizations, or those of the publisher, the editors and the reviewers. Any product that may be evaluated in this article, or claim that may be made by its manufacturer, is not guaranteed or endorsed by the publisher.
